# Dissociation of mitochondrial and ribosomal biogenesis during thallium administration in rat kidney

**DOI:** 10.1371/journal.pone.0311884

**Published:** 2024-12-04

**Authors:** Sho Sugahara, Kana Unuma, Shuheng Wen, Takeshi Funakoshi, Toshihiko Aki, Koichi Uemura

**Affiliations:** Department of Forensic Medicine, Graduate School of Medical and Dental Sciences, Tokyo Medical and Dental University (TMDU), Tokyo, Japan; Fujita Health University, JAPAN

## Abstract

Thallium (Tl) is a heavy metal with toxicity comparative to other heavy metals such as As, Cd, and Hg. Nevertheless, fewer studies have been reported concerning the molecular mechanism of Tl toxicity as compared to other heavy metals. To obtain insight into Tl toxicity in the kidney, rats were intraperitoneally administered Tl_2_SO_4_ (30 mg/kg), and the kidneys were removed 2 or 5 days later to examine the effects of Tl. Transcriptome analysis using DNA microarray of the rat kidney 2 and 5 days after Tl administration showed that cytoplasmic ribosomal proteins are the most upregulated category; many of the genes involved in ribosome biosynthesis were upregulated by Tl administration. This upregulation was associated with the activation of eukaryotic transcription initiation factor 2α (eIF2α), implying that increased ribosome biogenesis was linked to the subsequent activation of protein translation. In contrast, decreased mitochondrial biogenesis was revealed via proteomic analysis. Although we found an increase in Myc, a positive regulator of both ribosomal and mitochondrial biogenesis, decreased levels of NRF1 and TFAM, positive regulators of mitochondrial biogenesis whose gene expression is directory activated by Myc, were paradoxically observed. Taken together, differing responses of ribosomes and mitochondria to Tl toxicity were observed. Failure of transmission of the Myc signal to NRF1/TFAM might be involved in the observed disruption of coordinated responses in mitochondria and ribosomes during Tl administration in rat kidney.

## Introduction

Thallium (Tl) is a metal element found almost everywhere in the natural environment [1, 2]. Although there are relatively low concentrations of Tl in the natural environments, Tl contamination in beverages, tobaccos, and vegetables is becoming a big threat to human health [3]. Since Tl has applications in industries such as in semiconductors, Tl can be contaminated in air, water, and soils [3]. Tl ions can exist in two forms, Tl (I) and Tl (III), both of which have toxicities comparable to or higher than other heavy metals such as cadmium (Cd), mercury (Hg), and lead (Pb). The LD50 of thallium sulfate has been estimated to be around 30 mg/kg, 23.5 mg/kg, and 8–12 mg/kg in rats, mice, and humans, respectively [4, 5]. Symptoms of Tl intoxication include abdominal pain, paresthesia, etc., which are relatively commonly observed among other cases of drug intoxication [6]. In addition to public health concerns in which low and chronic Tl toxicity is involved, high and acute toxicity of this element plays crucial roles in fatal cases of Tl poisoning. Tl has a long history as the “poisoner’s poison” due to its tasteless, colorless, and odorless nature as well as extremely high toxicity [7, 8]. Difficulty in diagnosis and lack of effective antidotes have made Tl one of the most famous poisons used for not only homicides but also suicides. Tl was also used as a pesticide. Therefore, unintended ingestion of Tl and resultant severe poisoning including fatality have been still reported even in recent years [9–12]. Mechanistically, many TI toxicities derive from the similarities between potassium (K^+^) and Tl ions in terms of their radius and charge. Therefore, Tl disrupts essential cellular activities involving K^+^ ion such as Na^+^/Ka^+^ ATPase [1, 2]. The binding of TI to sulfhydryl groups of proteins and the subsequent protein inactivation is another mechanism of Tl toxicity [13, 14]. Due to its high solubility in water, Tl is easily absorbed into the human body through ingestion, inhalation, and the skin. Although to a lesser extent, Tl also can pass through the blood-brain barrier (BBB) and accumulate in the brain [15–17]. The tissue distribution of 16–48 mg/kg Tl_2_SO_4_ administered intraperitoneally to rats shows the highest accumulation in the kidney [17]. Nevertheless, there are few reports examining the cytotoxicity of Tl on kidney cells.

The cytotoxicity of Tl involves damage in an array of subcellular organelles, including mitochondria and ribosomes [1]. Like many other toxic chemicals harmful to the human body, mitochondrial damage is one of the major mechanisms of Tl toxicity. Mitochondria are dynamic organelles undergoing continuous fission and fusion [18]. Mitochondrial biogenesis, the self-replication through the fission of pre-existing mitochondria, is a phenomenon observed during periods of increased demand for cellular energy such as during aerobic excise [18]. Nuclear respiratory factor 1 (NRF1) and mitochondrial transcription factor A (TFAM) are transcription factors that play crucial roles in mitochondrial biosynthesis [19]. For example, although both NRF1 and TFAM are nuclear-encoded genes, NRF1 activates nuclear-encoded mitochondrial electron transfer chain (ETC) genes directly while mitochondria-encoded ETC genes are activated indirectly through the activation of TFAM [20]. Ribosomes are another organelle shown to be damaged by Tl [21, 22]. In contrast to mitochondrial biogenesis, ribosome biogenesis is achieved through the construction of complete ribosome particles from their parts, ribosomal RNAs and proteins [23, 24]. Since ribosomes are highly energy consuming organelles, ribosome biogenesis is almost always accompanied by mitochondrial biogenesis [25].

c-Myc (or Myc) is an oncogene that fulfills its tumor-promoting roles by working as a transcription factor [26]. Myc promotes cell cycle progression through the upregulation of cyclins and the downregulation of cyclin-dependent protein kinase inhibitors (CDKIs) [26]. In accordance with the fact that cell proliferation increases the cellular demand for energy, such as ATP, and biomaterials, such as proteins, it has been demonstrated that both ribosomal and mitochondrial biogenesis are positively regulated by Myc [27–29]. Although the simultaneous activation of cell cycle progression, mitochondrial biogenesis, and ribosomal biogenesis is matched well to the pathophysiology of cancer, little is known about the status and roles of these cellular events in the pathophysiology of Tl intoxication. To gain insight into the molecular mechanism of Tl toxicity in the kidney, we found that there is a disturbance in the balance among these three cellular responses to Tl toxicity.

## Experimental procedures

### Materials

Thallium sulfate (Tl_2_SO_4_) was purchased from WAKO pure chemicals (Osaka, Japan). All other reagents are also commercially available.

### Ethics statement

All methods were carried out in accordance with relevant guidelines and regulations. All animal experiments were approved by the Institutional Animal Care and Use Committee of Tokyo Medical and Dental University (approval No. A2023-080C) and reported in accordance with Animal Research: Reporting of In Vivo Experiments (ARRIVE) guidelines and regulations. To alleviate suffering, we adopted humane endpoints such as decreased body weight, reduced consumption of food/water, and behavioral changes.

### Animals

Wistar rats (7-weeks-old, male) were purchased from Oriental Yeast Co. Ltd. (Tokyo, Japan). Rats were housed in groups of 2 animals in specific-pathogen-free conditions and kept under standardized conditions including bedding materials and free access to food and water, an atmosphere at 25°C, and a 12 h light/dark cycle throughout the experiments. To investigate the effects of sub-acute Tl exposure on the kidney, they were randomly divided into 3 groups: control (n = 4), Tl (2 days, n = 4), and Tl (5 days, n = 5) groups. Since we intended to investigate the effects of the half lethal dose of Tl on the kidney and the reported LD50 of Tl for rats is 30 mg/kg, they were intraperitoneally administrated double distilled water or Tl_2_SO_4_ (30 mg/kg), which was achieved by an administration of 1.7 mL solution per 250 g (body weight). The solution was prepared fresh just before the injection, which lasted approx. for 1 min for each rat. Rats were monitored for health every day. Although humane endpoints including decreased body weights, reduced consumption of food/water, and behavioral changes were not observed, 2 rats died 3–4 days after Tl administration. They were included in the Tl (5 days) group. Two or five days after the Tl_2_SO_4_ administration, the rats were sacrificed by an overdose of sodium pentobarbital (40 mg/kg), and the kidneys were excised. Excised tissues were divided into 4 pieces and stored at -80°C for extraction of RNA and protein, 4°C in 4% PFA solution for immunohistochemistry, and phosphate buffer for electron microscopy. Investigators were not blinded to sample information. Results considered to be having experimental errors were excluded.

### DNA microarray and qPCR analysis

Total RNA was extracted from the rat kidney using Trizol reagent (Thermo). Tissues frozen at -80°C were thawed by leaving them at room temperature for approx. 1 hour. Total RNA was extracted from the tissues using Trizol (Thermo) according to manufacturer’s instruction. The concentration and purity of RNA were determined by NanoDrop One (Thermo) by measuring A_260_ and A_260_/A_280_, respectively. For DNA microarray analysis, total RNA was further purified using an RNeasy RNA purification kit (QIAGEN). Total RNA was examined for its integrity by a BioAnalyzer (Agilent) and hybridized to a ClariomS array (Thermo). The results were deposited in the GEO database (https://www.ncbi.nlm.nih.gov/geo/, accession number GSE269635). Transcriptome Analysis Console software (TAC, Thermo) was used to analyze the results of DNA microarray analysis. For qPCR analysis, cDNA synthesis was performed using 50 U of SuperScriptII reverse transcriptase (Thermo), 0.5 μg of oligo(dT)_15_, 1 μg of total RNA, and 0.4 mM dNTPs in a 20 μL of reaction buffer [50 mM Tris (pH 8.3), 70 mM KCl, 3 mM MgCl_2_, and 10 mM DTT] at 42°C for 50 min., followed by 70°C for 15 min. cDNA obtained was stored at -20°C until use. A StepOnePlus PCR machine and StepOne Software (ver.2.3. Thermo) were used to perform quantitative reverse transcription-mediated real time PCR (qPCR) and analyze the data, respectively. A comparative Cq (also referred to as Ct) method (2^-ΔΔCq^ method) [30] and SYBR Green fluorescence dye (GoTaq qPCR master mixture, Promega, Madison, MI) were used to calculate the relative abundance of RNA and to detect amplification products, respectively. The PCR condition was 95°C for 20 s, followed by 40 cycles of 95°C for 3 s and 60°C for 30 s. The primers used are listed in S1 Table in S1 File. After the cycle of PCR, the melt curve analysis was performed with the condition of 95°C for 15 s, followed by 60–95°C to confirm the specific amplification of the PCR product.

### MALDI-TOF-MS

Matrix Assisted Laser Desorption/Ionization-Time Of Flight-Mass Spectrometry (MALDI-TOF-MS) analysis was performed as described previously [31]. In brief, gel slices excised from CBB-stained gels were treated with DTT and iodoacetamide to reduce disulfide bonds and modify cysteine residues of the proteins within the gel slices. After digestion with trypsin, the resultant peptides were subjected to MALDI-TOF-MS (UltrafleXtreme, Bruker Daltonics, Billerica, MA, USA) analysis using α-cyano-4-hydroxycinnamic acid (Bruker Daltonics) as the matrix.

### Immunoblot analysis

Immunoblot analysis was performed as described previously [31, 32]. In brief, kidney lysates were separated by SDS-PAGE, blotted to a PVDF membrane, and probed with antibodies (S2 Table in S1 File). Antigens were visualized using peroxidase-conjugated anti-IgG secondary antibodies and ECL reagents. All blots with membrane visible are provided in S1 Fig in S1 File. We sometimes cut membranes horizontally and exposed to different antibodies. Optical densities of test as well as control proteins were determined by use of a densitometric program (CS Analyzer ver. 4, ATTO) (S3 Table in S1 File). Then, relative levels of test proteins were determined by dividing the densities of test proteins by that of control proteins.

### Immunohistochemistry

Immunohistochemical (IHC) analysis was performed as described [32]. Paraffin-embedded sections were deparaffinized and antigenicity was retrieved through microwave heating. The sections were incubated at 4°C overnight with 1/100 diluted rabbit anti-RPS3 antibody (S2 Table in S1 File). A Histofine Simple Stain Max PO (MULTI) kit (Nichirei Biosciences Inc., Tokyo, Japan) was used to visualize antigens. Diaminobenzidine (DAB) was used as a substrate.

### Electron microscopy

Electron microscopy of the rat kidney was performed as described [32]. Briefly, tissue sections were fixed with 4.5% paraformaldehyde and 2.5% glutaraldehyde in phosphate buffer, treated with 1% osmium tetroxide, dehydrated with ethanol, and embedded in Epon epoxy resin. The sections were sliced into ultrathin sections (90 nm thickness) and stained with uranyl acetate and lead citrate. Transmission EM analysis was performed using a Hitachi H-7100 (Hitachinaka, Japan).

### Statistical analysis

After ANOVA as the pre hoc test, Dunnett’s post hoc test was used and p<0.05 is considered statistically significant.

## Results

### Pathohistological and transcriptome analysis of kidney from rats administered Tl

**Fig 1:**
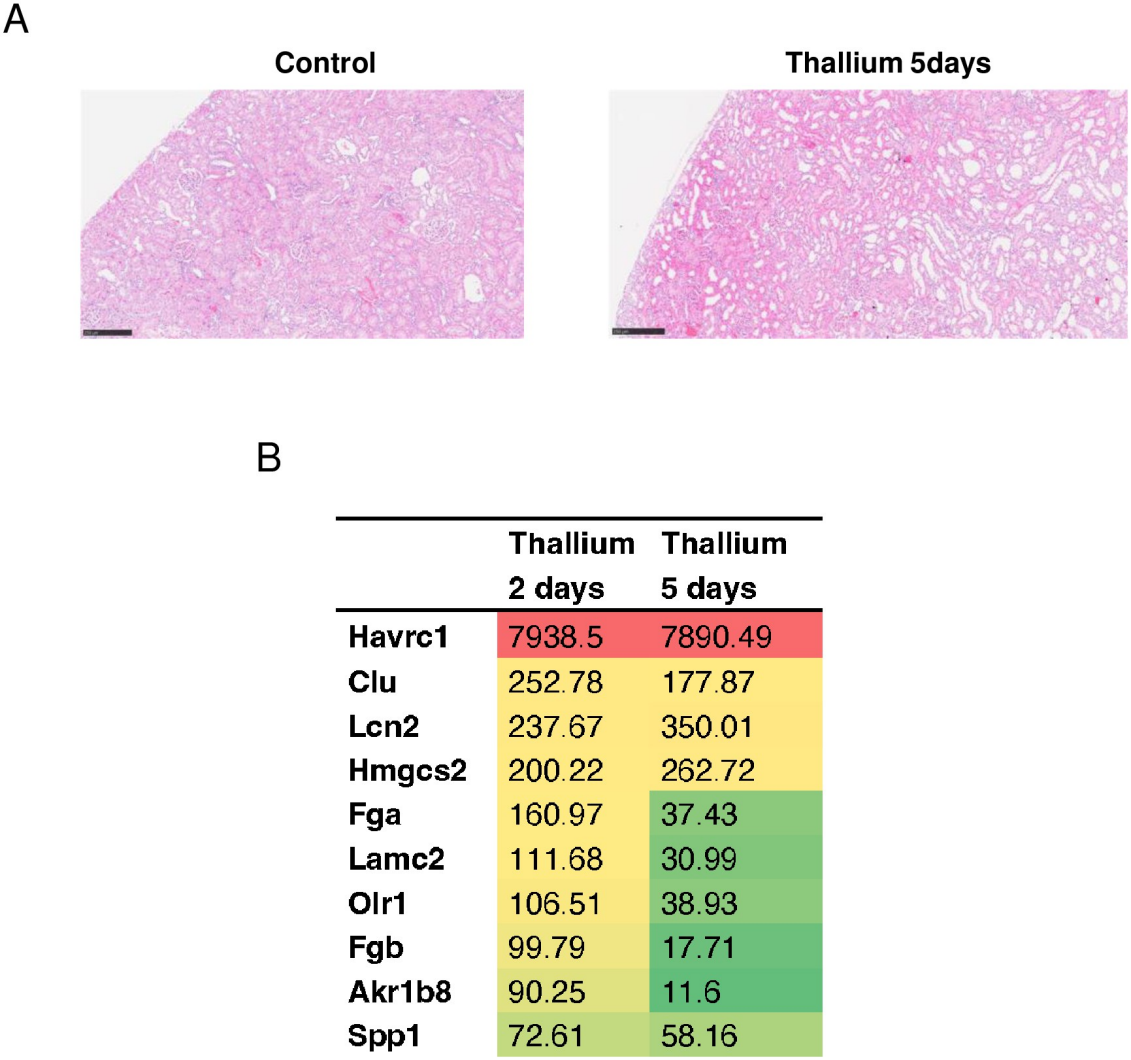
Tl damages rat kidney. Rats were administrated a single dose of double distilled water or 30 mg/kg Tl. Five days after the administration of Tl or double distilled water (Control), kidneys were excised and subjected to H&E stain (A). Bar, 250 μm. Total RNA was also extracted from the kidneys of rats (Tl 2 days) and subjected to DNA microarray analysis (B). TOP10 genes most induced by Tl (2 days) and fold increases relative to control group are shown.

We first evaluated whether Tl damages rat kidney or not. H&E stain of the kidney from rats administered Tl (30 mg/kg, 5 days) showed mild congestion, flattened tubular epithelial cells, tubular dilatation, and widespread sloughing of tubular cells (Fig 1A). Thus, we concluded that the experimental setting adopted to this study indeed damaged rat kidney. We next investigated differences in the expressed genes in rat kidney from the Tl and control groups. Transcriptome analysis using DNA microarray showed that the most increased gene in response to Tl administration was hepatitis A virus cellular receptor 1 (Havcr1), which is also known as kidney injury molecule 1 (Kim1) and is considered a marker of kidney injury [33–35] (Fig 1B). Furthermore, other genes suggested as possible markers of kidney injury, such as clusterin (Clu) and lipocalin 2 (Lcn2), were also included in the list of highly upregulated genes [36, 37] (Fig 1B). Thus, the results of transcriptome analysis confirmed that the administration of Tl at a dose of 30 mg/kg results in the development of kidney injuries 2 and 5 days after administration. It was also demonstrated by the transcriptome analysis that in the kidneys of rats administered Tl, ribosome biogenesis was the most influenced category (Table 1).

**Table 1:** TOP10 categories most influenced by Tl in the kidney of rats.

2 days after Tl administration			
Categories	Significance	Up	Down
Cytoplasmic ribosomal proteins	12.12	34	0
Cell cycle	11.36	31	2
G1 to S cell cycle control	11.27	26	2
Spinal cord injury	6.37	21	7
DNA replication	6.29	16	0
Adipogenesis	4.61	16	13
Focal adhesion	4.13	30	7
TGF-beta signaling pathway	3.49	10	4
Alpha 6 beta 4 integrin signaling pathway	3.32	14	2
Senescence and autophagy	3.17	17	5

Up: number of the genes upregulated more than 2-fold

Down: number of the genes downregulated more than 2-fold

### Upregulation of a ribosomal protein and activation of a translation initiation factor in kidneys from rats administered Tl

**Fig 2:**
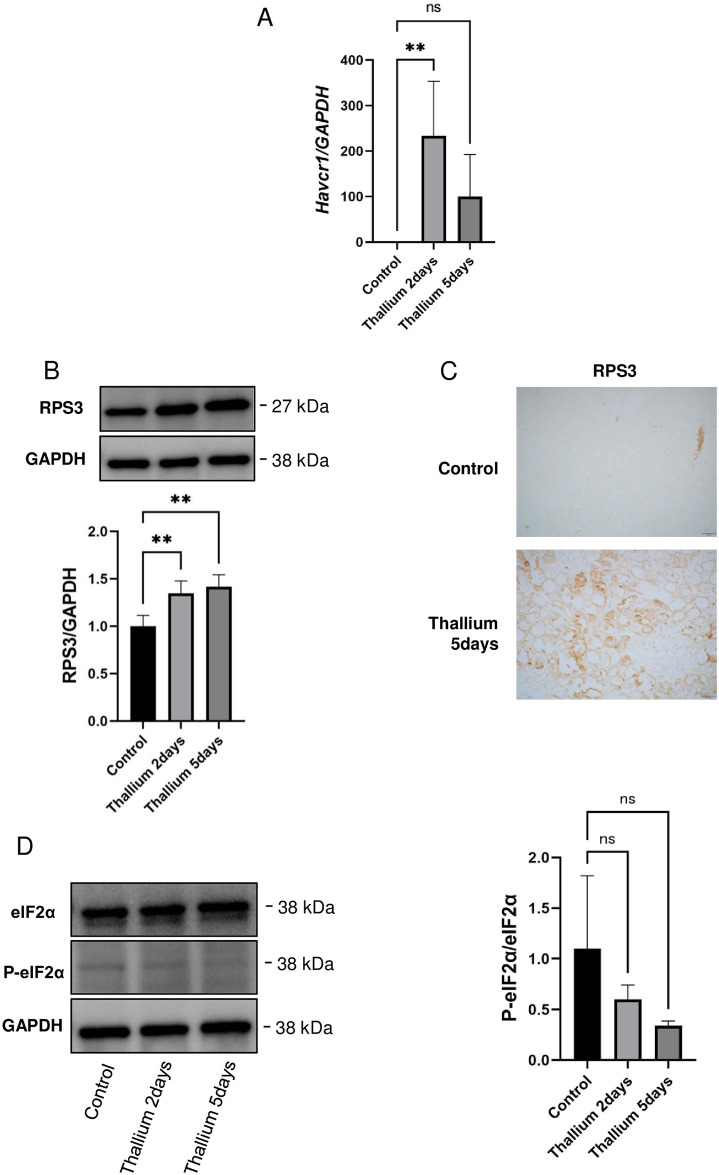
Increased biogenesis of ribosomes in the kidneys from rats administered Tl. Rats were administrated a single dose of double distilled water or 30 mg/kg Tl. Two or 5 days after the administration of Tl or double distilled water (Control), kidneys were excised and subjected to the analysis. (A) qPCR analysis of *Havcr1* gene expression. (B) Immunoblot analysis of RPS3. GAPDH served as an internal standard. (C) IHC analysis of RPS3. (D) Immunoblot analysis of total and ser-51 phosphorylated eIF2α (eIF2α and p-eIF2α). GAPDH served as an internal standard. Graphs show mean and S.D. (n = 4 in control and Tl 2 days groups and n = 3 in Tl 5 days group.). Two samples from Tl 5 days group were excluded due to technical errors. In each graph, the mean value of the control group was set to 1. *, p<0.05, **, p<0.01 versus control by Dunnett’s post hoc test.

As shown in Fig 2A, the Tl-induced increase in Havcr1 was confirmed by qPCR. To confirm the increase in ribosomal proteins, immunoblot analysis of ribosomal protein S3 (RPS3), which is a subunit of the 40S ribosome and commonly used as a ribosomal marker [23, 38], was performed. As shown in Fig 2B, the levels of RPS3 were increased in the Tl groups as compared to the control group. IHC analysis of RPS3 further confirmed that the number of ribosomes in the Tl groups was increased as compared to the control group (Fig 2C). To evaluate whether the increased expression of ribosomal genes observed in Tl-administered rat kidney was accompanied by an increase in protein synthesis, we evaluated the phosphorylation status of eukaryotic translation initiation factor 2α (eIF2α); eIF2α is required for translation initiation and the activity of eIF2α has been shown to be suppressed by phosphorylation on ser-51 [39, 40]. As shown in Fig 2D, the levels of ser-51 phosphorylated eIF2α were decreased in the Tl groups as compared to the control group. These results show that Tl increases ribosomal gene expression, which should be followed by increased protein synthesis.

### Identification of MDH2 as a protein whose expression is severely decreased by Tl

**Fig 3:**
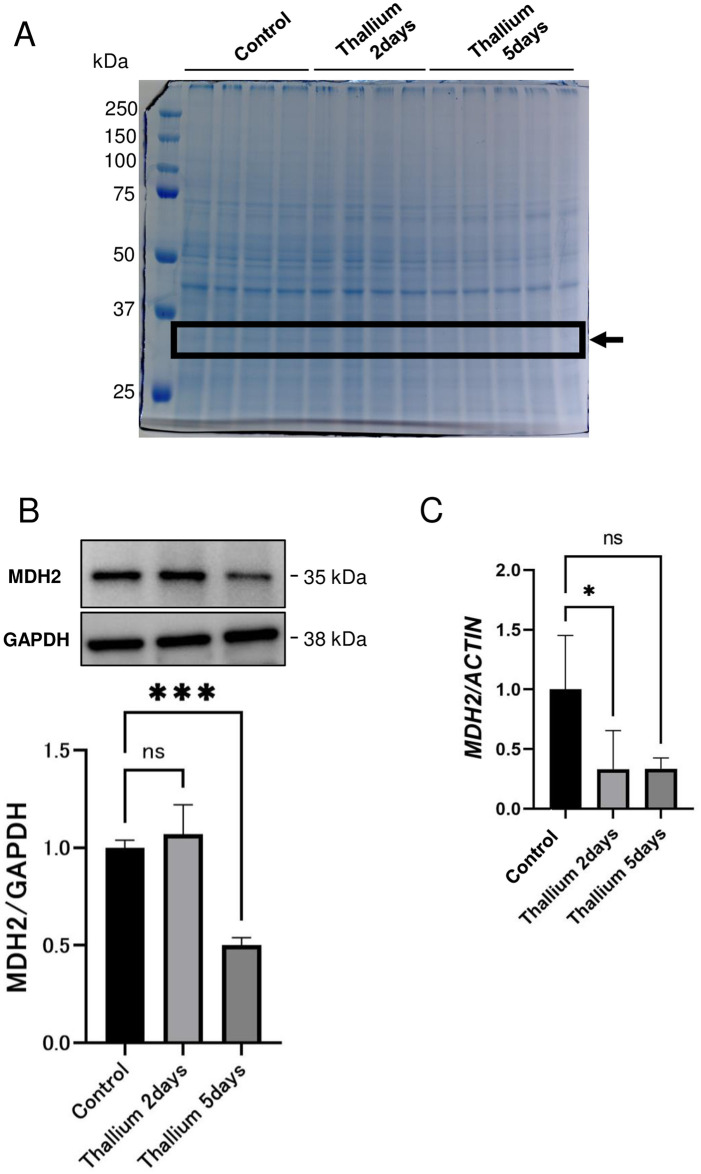
Identification of MDH2 as a protein whose expression is regulated by Tl. Rats were administrated a single dose of double distilled water or 30 mg/kg Tl. 2 or 5 days after the administration of Tl or double distilled water (Cont.), kidneys were excised and subjected to the analysis. (A) CBB stained gel of kidney lysates. The band marked with an arrow was excised from the gel and identified as MDH2 by MALDI-TOF-MS. (B) Immunoblot analysis of MDH2. GAPDH served as an internal control. (C) qPCR analysis of *MDH2* gene expression. Actin served as an internal control. Graphs show mean and S.D. (n = 4 in control and Tl 2 days groups and n = 3 in Tl 5 days group). Two samples from Tl 5 days group were excluded due to technical errors. In each graph, the mean value of the control group was set to 1. *, p<0.05, ***, p<0.001 versus control by Dunnett’s post hoc test.

We further applied MALDI-TOF-MS analysis as a method of proteomics to identify differentially expressed proteins before and after Tl treatment. CBB staining of kidney lysates revealed that a protein with a molecular mass of approximately 35 kDa was decreased by Tl treatment (Fig 3A). MALDI-TOF-MS analysis identified this protein as malate dehydrogenase 2 (MDH2), an enzyme involved in the TCA cycle within mitochondria [41]. Immunoblot analysis confirmed the decrease of MDH2 in response to Tl treatment (Fig 3B). Interestingly, qPCR analysis also confirmed the decrease in MDH2, suggesting that the decrease occurred at the transcriptional level (Fig 3C).

### Decreased mitochondrial biogenesis in kidneys from rats administered Tl

**Fig 4:**
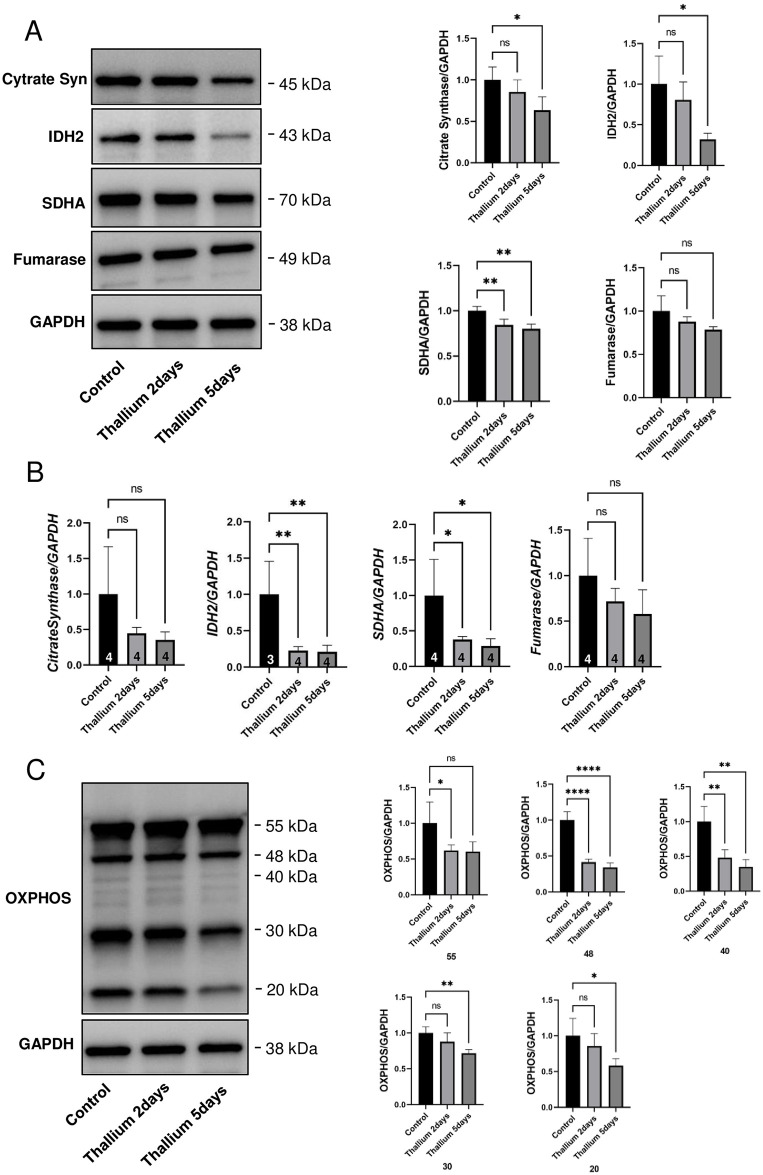
Decreased expression of TCA cycle and ETC enzymes in kidneys from rats administered Tl. Rats were administrated a single dose of double distilled water or 30 mg/kg Tl. 2 or 5 days after the administration of Tl or double distilled water (Cont.), kidneys were excised and subjected to the analysis. (A) Immunoblot analysis of TCA cycle enzyme gene expression. GAPDH served as an internal control. (n = 4 in control and Tl 2 days groups and n = 3 in Tl 5 days group). Graphs show mean and S.D. (n = 4 in control and Tl 2 days groups and n = 3 in Tl 5 days group). Two samples from Tl 5 days group were excluded due to technical errors. (B) qPCR analysis of TCA cycle enzyme gene expression. GAPDH served as an internal control. Numbers in the bars indicate sample size. Several samples were excluded due to technical errors. (C) Immunoblot analysis of OXPHOX proteins (55, CV-ATP5A-55kDa; 48, CIII-UQCRC2-48 kDa; 40, CIV-MTCO1-40 kDa; 30, CII-SDHB-30 kDa; 20, CI-NDUFB8-20 kDa). GAPDH served as an internal control. Graphs show mean and S.D. (n = 4 in control and Tl 2 days groups and n = 3 in Tl 5 days group). Two samples from Tl 5 days group were excluded due to technical errors. In each graph, the mean value of the control group was set to 1. *, p<0.05, **, p<0.01, ****, p<0.0001 versus control by Dunnett’s post hoc test.

We further examined whether the Tl-induced decrease in MDH2 is specific to this protein or not. Immunoblot analysis showed that all other TCA cycle enzymes examined [citrate synthase, isocitrate dehydrogenase 2 (IDH2), succinate dehydrogenase complex flavoprotein subunit A (SDHA), fumarase] were decreased after Tl treatment (Fig 4A). qPCR analysis also confirmed the transcriptional downregulations of these four genes (Fig 4B) In addition, immunoblot analysis of OXPHOS proteins revealed that many ECT proteins were also decreased by Tl treatment (Fig 4C), suggesting that the relative abundance of mitochondria should be decreased by Tl treatment. These results revealed that, instead of the increased synthesis of ribosomal proteins, mitochondrial biogenesis was decreased by Tl treatment.

### EM analysis of kidneys from rats administered Tl

**Fig 5:**
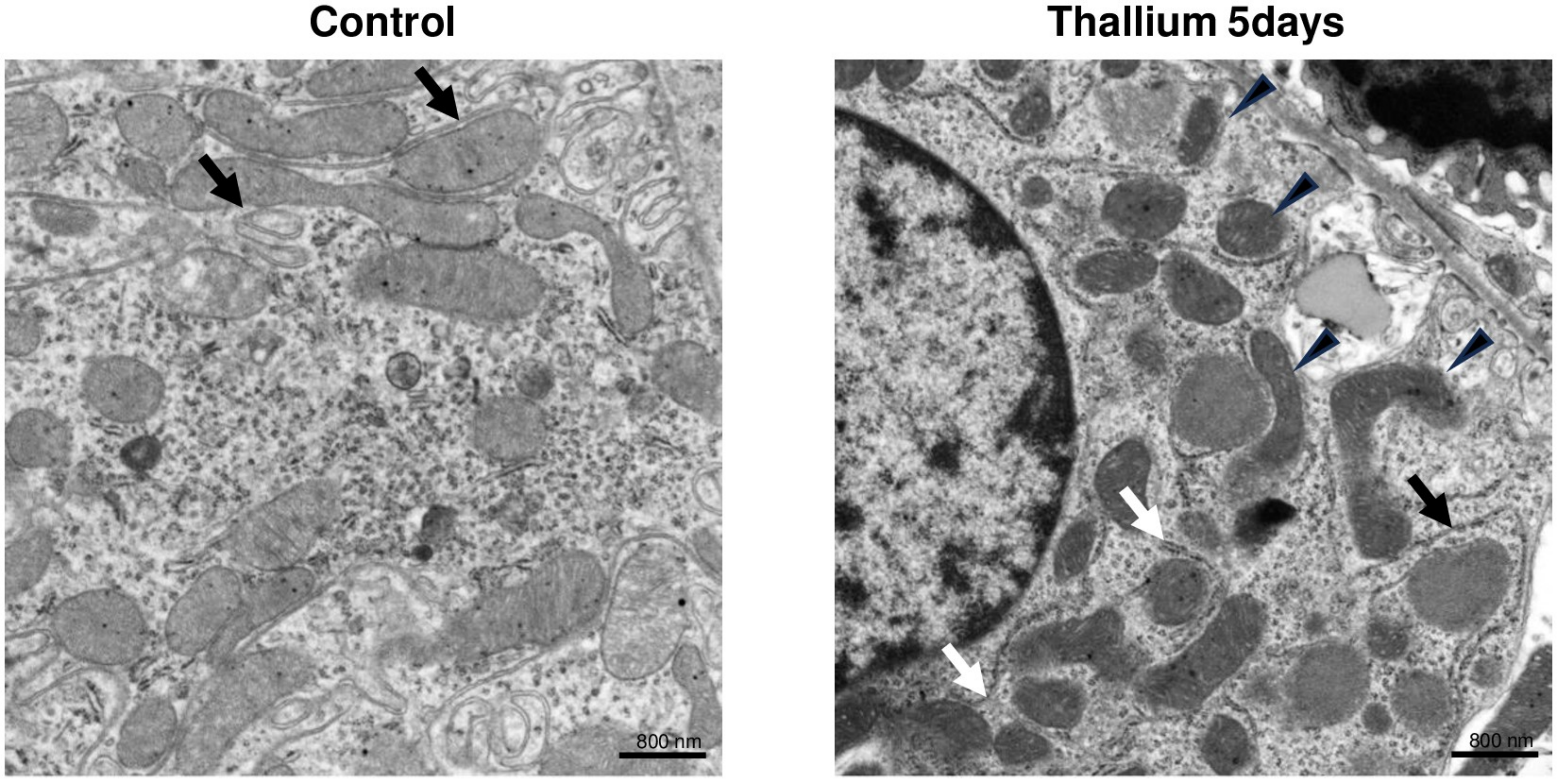
Electron microscopic analysis of kidneys from rats administered Tl. Rats were administrated a single dose of double distilled water or 30 mg/kg Tl. 2 or 5 days after the administration of Tl or double distilled water (Cont.), kidneys were excised and subjected to the analysis. Transmission electron microscopic images of kidneys. Magnification, x10,000. Black and white arrows indicate smooth and rough ER, respectively. Arrowheads indicate electron-dense mitochondrion.

To further confirm the differential effects of Tl on ribosomal and mitochondrial biosynthesis, transmission EM analysis was conducted. As shown in Fig 5, there were numerous opaque mitochondria, which seemed to be electron-dense due to their dysfunction, in the Tl group. Furthermore, a higher relative abundance of rough endoplasmic reticulum (ER) was observed in the Tl group as compared to the control group (Fig 5). These results confirm our finding that Tl increases synthesis of ribosomal genes/proteins (Fig 2) while it decreases mitochondrial biosynthesis/regeneration (Fig 4).

### Increased levels of Myc do not lead to the activation of the NRF1/TFAM pathway of mitochondrial biogenesis in the kidneys from rats administered Tl

**Fig 6:**
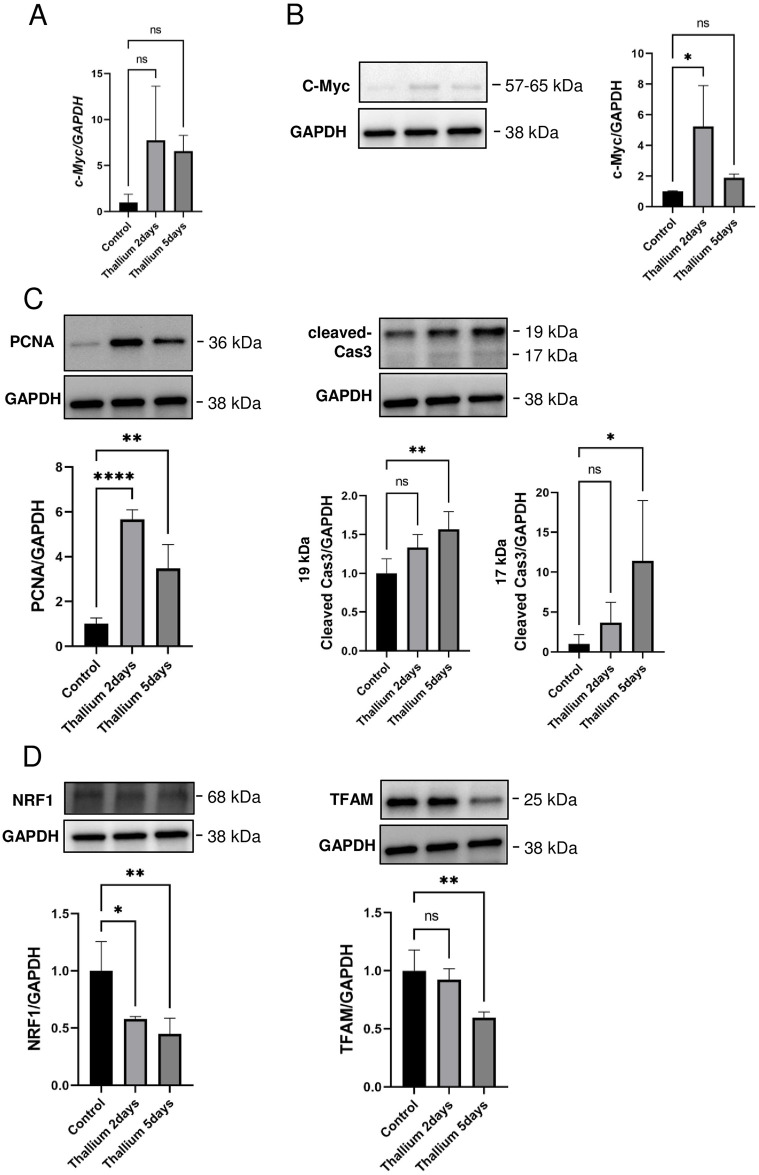
Respective increase and decrease in Myc and NRF1/TFAM in kidneys from rats administered Tl. Rats were administered a single dose of double distilled water or 30 mg/kg Tl. 2 or 5 days after the administration of Tl or double distilled water (Cont.), kidneys were excised and subjected to the analysis. (A) qPCR analysis of Myc gene expression. GAPDH served as an internal control. (B-D) Immunoblot analysis of c-Myc (B), PCNA (C), cleaved-caspase 3 (C), and NRF1/TFAM (D) protein levels. GAPDH served as an internal control. Graphs show mean and S.D. (n = 4 in control and Tl 2 days groups and n = 3 in Tl 5 days group). Two samples from Tl 5 days group were excluded due to technical errors. In each graph, the mean value of the control group was set to 1. **, p<0.01, ****, p<0.0001 versus control by Dunnett’s post hoc test.

There are several transcription factors reported to be involved in the biosynthesis of these organelles. Among them, we focused on Myc, since our transcriptome analysis indicated a massive increase in Myc gene expression in response to Tl (25.32 and 20.13-fold increases 2 and 5 days after Tl administration, respectively). We checked Myc gene expression via qPCR analysis and confirmed that Myc expression was indeed increased in the Tl groups (Fig 6A), which was further confirmed by immunoblot analysis (Fig 6B) Furthermore, our transcriptome analysis also indicated that Tl transiently increased the expression of proliferating cell nuclear antigen (PCNA), a marker of proliferating cells [42] (4.44 and 1-fold increases 2 and 5 days after Tl administration, respectively)). We checked PCNA protein levels and confirmed the transient increase of PCNA following Tl administration (Fig 6C). Since Myc can induce apoptosis [43], we also examined caspase3 activation in kidneys from Tl-administered rats, and observed significant amounts of apoptosis in the Tl groups (Fig 6C). Taken together, the Tl-induced increase in Myc should lead to increased expression of ribosomal genes and the proliferation of kidney cells, which might indicate cellular regeneration to compensate for apoptotic cell loss. Indeed, it has been demonstrated that several types of kidney cells still have the capacity to proliferate [44]. We finally examined the levels of nuclear respiratory factor 1 (NRF1) and mitochondrial transcription factor A (TFAM), both of which play important roles in the biogenesis of mitochondria with NRF1 having been shown to be a direct target of Myc [19]. As shown in Fig 6D, both NRF1 and TFAM were decreased in the kidneys from rats administered Tl. These results coincide well with the observation that Tl decreases the relative abundance of mitochondrial proteins (Fig 4). The result also raises the possibility that Myc activation may not be transmitted to NRF1/TFAM, in contrast to the observation that both cell proliferation and expression of ribosomal genes are properly activated in response to Myc activation.

## Discussion

Although Tl elicits various cellular injuries such as DNA damage [1] and ER stress [45], acute Tl toxicity on multiple organs is generally considered derived from oxidative stress. For example, it has been demonstrated that acute Tl toxicity on multiple organs in rats can be attenuated by not only metal-binding metallothionein but also Prussian blue, an anti-oxidative reagent [46–48]. In addition to these studies examining the mechanism of Tl toxicity in animal models, recent research about Tl toxicity on human has shown that kidney is an important target organ of Tl poisoning. For example, it has been shown that low concentrations of Tl can induce early damage to kidney, liver, and myocardium in children [17, 49]. This study should suggest that kidney is one of the earliest target organs of Tl toxicity both in human and rats. Rayisyan et al. examined a total of 39 patients suffering from severe Tl poisoning and showed that in addition to standard therapy against Tl poisoning such as Prussian blue, potassium-iron hexacyanoferrate and an iron chelator Deferasirox showed additive effects on the recovery from liver and kidney injuries caused by Tl [50]. Our current results demonstrating a dissociation between ribosomal and mitochondrial responses to Tl might suggest a novel mechanism of Tl cytotoxicity as well as provide more clues to possible therapy against Tl poisoning.

In contrast to our current results, it has been demonstrated that Tl inactivates ribosomal function both in vitro and in vivo [21, 22]. Detailed analysis using HEK293 human embryonic kidney cells by Chou et al. demonstrated that Tl reduced specifically the amounts of 60S ribosome [22]. They also showed that the phenomenon observed was not due to increased degradation of 60S ribosome but to the suppression of the biosynthesis of 60S ribosome [22]. They also showed a decrease in the levels of RPS3 in response to Tl administration [22]. Totally opposite to the results of that report, we observed increased expression of genes for ribosomal biogenesis (Table 1). Indeed, we observed increased levels of RPS3 in response to Tl administration both with immunoblot and IHC analysis (Fig 2). Furthermore, an increased abundance of ribosomes should be connected to an increase in protein synthesis, as eIF2 was found to be activated by Tl (Fig 2). Currently, we have no logical way to explain the discrepancy between our results and those of Chou et al. [22]. Since ribosomal impairment is often followed by their regeneration achieved by increased ribosome biogenesis, there might be possible that our current results represent the regeneration phase of ribosome homeostasis during Tl administration. Nevertheless, this is the first report to the best of our knowledge, demonstrating that Tl can increase ribosomal gene expression, at least in some experimental settings.

Our another result that mitochondrial biogenesis should be decreased in response to Tl administration coincides well with the fact that Tl inhibits mitochondrial functions. It has been reported that Tl impairs mitochondria by inhibiting mitochondrial Na^+^/Ka^+^-ATPase and ETC enzymes harboring sulfhydryl resides essential for enzymatic function but that can be a target of Tl binding. In addition to these reports, our results provide evidence of not only mitochondrial dysfunction (Fig 5) but also the impairment of mitochondrial biogenesis (Figs 4 and 6). Indeed, we demonstrated decreases in both TCA cycle and ETC enzymes, which should be the results of transcriptional suppression rather than degradation. In accordance with the suppression of transcription, we also observed decreased levels of TFAM and NRF1, confirming that mitochondrial biogenesis should decrease in response to Tl in our experimental setting. These observations might be surprising since ribosomal and mitochondrial biogenesis should occur simultaneously due to the fact that ribosomes are highly energy-consuming organelles and, therefore, require healthy mitochondria to provide cellular energy such as in the form of ATP. In accordance with the fact that ribosomal and mitochondrial biogenesis should occur in parallel, we observed Myc activation (Fig 6). Myc is a transcriptional factor involved in the biogenesis of both these organelles. However, we did not observe any corresponding activation of NRF1/TFAM, target genes of Myc. Lin et al. showed that HIF1α signaling suppresses the Myc-TFAM axis [51]. Thus, therapeutic intervention, such as by the suppression of HIF1α, might be able to correct the failure of Myc signaling to mitochondrial biogenesis. Further study might lead to ways to achieve the simultaneous occurrence of mitochondrial and ribosomal biogenesis, which should strongly enhance the possibility of kidney cell recovery from Tl toxicity.

## Supporting information

S1 File (ZIP)
