# Supplementary material for: Dissociation of mitochondrial and ribosomal biogenesis during thallium administration in rat kidney
Source: PLoS One. 2024 Dec 4;19(12):e0311884. doi: 10.1371/journal.pone.0311884 (PMC11616847; doi:10.1371/journal.pone.0311884)
Supplement: S1 File — (ZIP) [file pone.0311884.s001.zip › Supporting information/S2 Table.pptx]

## Slide 1
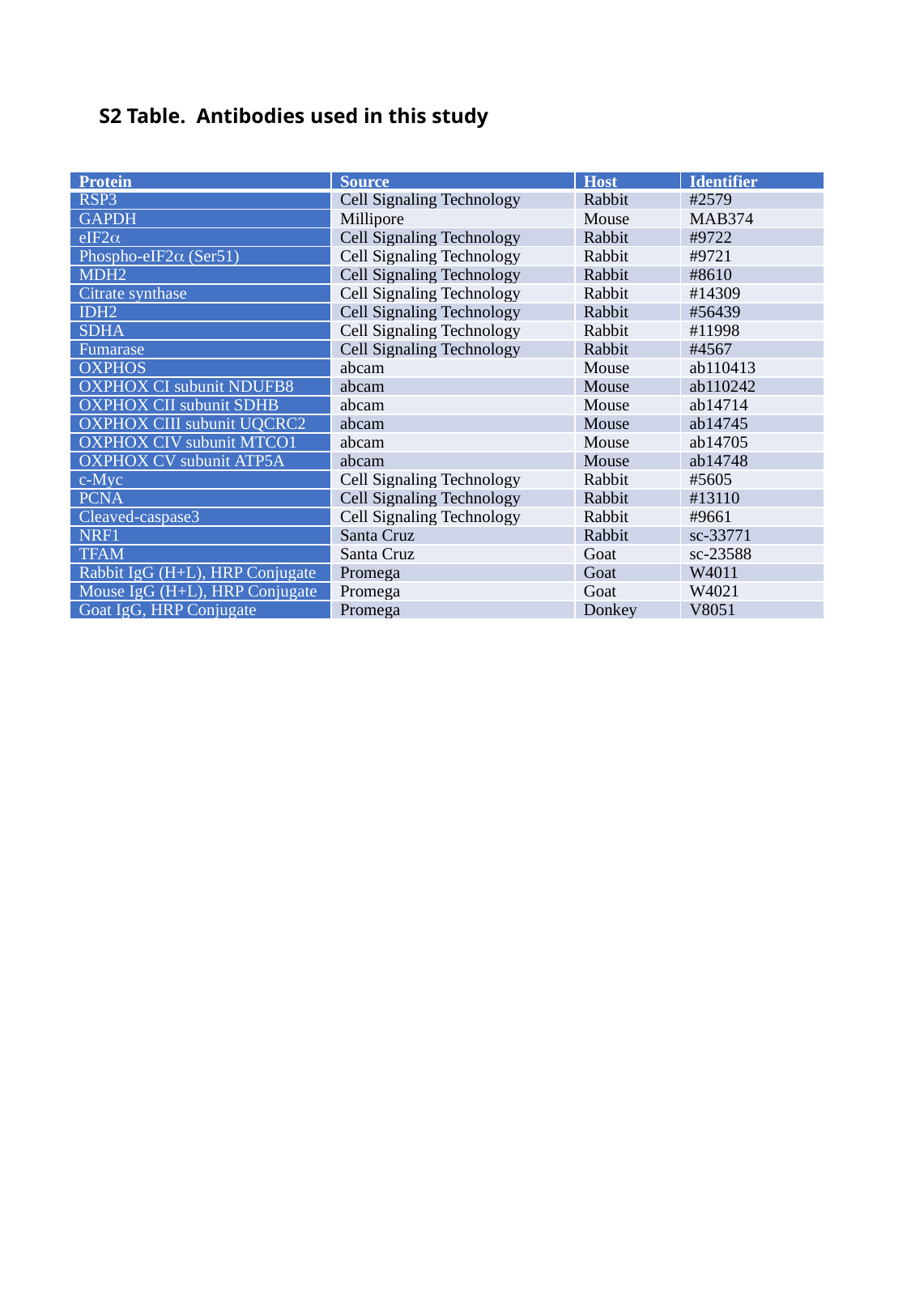

S2 Table. Antibodies used in this study
| Protein | Source | Host | Identifier |
| --- | --- | --- | --- |
| RSP3 | Cell Signaling Technology | Rabbit | #2579 |
| GAPDH | Millipore | Mouse | MAB374 |
| eIF2a | Cell Signaling Technology | Rabbit | #9722 |
| Phospho-eIF2a (Ser51) | Cell Signaling Technology | Rabbit | #9721 |
| MDH2 | Cell Signaling Technology | Rabbit | #8610 |
| Citrate synthase | Cell Signaling Technology | Rabbit | #14309 |
| IDH2 | Cell Signaling Technology | Rabbit | #56439 |
| SDHA | Cell Signaling Technology | Rabbit | #11998 |
| Fumarase | Cell Signaling Technology | Rabbit | #4567 |
| OXPHOS | abcam | Mouse | ab110413 |
| OXPHOX CI subunit NDUFB8 | abcam | Mouse | ab110242 |
| OXPHOX CII subunit SDHB | abcam | Mouse | ab14714 |
| OXPHOX CIII subunit UQCRC2 | abcam | Mouse | ab14745 |
| OXPHOX CIV subunit MTCO1 | abcam | Mouse | ab14705 |
| OXPHOX CV subunit ATP5A | abcam | Mouse | ab14748 |
| c-Myc | Cell Signaling Technology | Rabbit | #5605 |
| PCNA | Cell Signaling Technology | Rabbit | #13110 |
| Cleaved-caspase3 | Cell Signaling Technology | Rabbit | #9661 |
| NRF1 | Santa Cruz | Rabbit | sc-33771 |
| TFAM | Santa Cruz | Goat | sc-23588 |
| Rabbit IgG (H+L), HRP Conjugate | Promega | Goat | W4011 |
| Mouse IgG (H+L), HRP Conjugate | Promega | Goat | W4021 |
| Goat IgG, HRP Conjugate | Promega | Donkey | V8051 |
