# Supplementary material for: Dissociation of mitochondrial and ribosomal biogenesis during thallium administration in rat kidney
Source: PLoS One. 2024 Dec 4;19(12):e0311884. doi: 10.1371/journal.pone.0311884 (PMC11616847; doi:10.1371/journal.pone.0311884)
Supplement: S1 File — (ZIP) [file pone.0311884.s001.zip › Supporting information/S1 Table.pptx]

## Slide 1
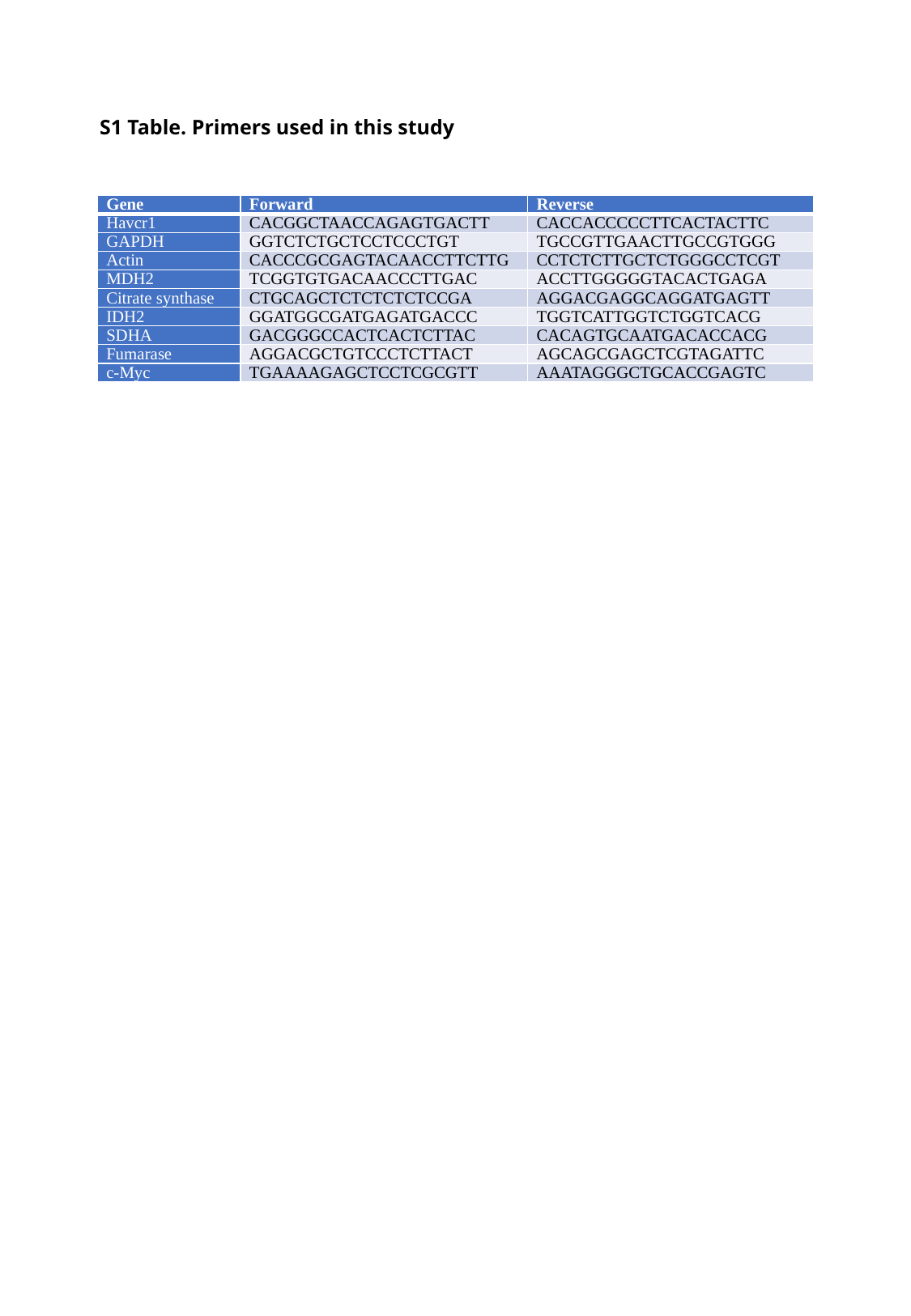

S1 Table. Primers used in this study
| Gene | Forward | Reverse |
| --- | --- | --- |
| Havcr1 | CACGGCTAACCAGAGTGACTT | CACCACCCCCTTCACTACTTC |
| GAPDH | GGTCTCTGCTCCTCCCTGT | TGCCGTTGAACTTGCCGTGGG |
| Actin | CACCCGCGAGTACAACCTTCTTG | CCTCTCTTGCTCTGGGCCTCGT |
| MDH2 | TCGGTGTGACAACCCTTGAC | ACCTTGGGGGTACACTGAGA |
| Citrate synthase | CTGCAGCTCTCTCTCTCCGA | AGGACGAGGCAGGATGAGTT |
| IDH2 | GGATGGCGATGAGATGACCC | TGGTCATTGGTCTGGTCACG |
| SDHA | GACGGGCCACTCACTCTTAC | CACAGTGCAATGACACCACG |
| Fumarase | AGGACGCTGTCCCTCTTACT | AGCAGCGAGCTCGTAGATTC |
| c-Myc | TGAAAAGAGCTCCTCGCGTT | AAATAGGGCTGCACCGAGTC |
