# Supplementary material for: Dissociation of mitochondrial and ribosomal biogenesis during thallium administration in rat kidney
Source: PLoS One. 2024 Dec 4;19(12):e0311884. doi: 10.1371/journal.pone.0311884 (PMC11616847; doi:10.1371/journal.pone.0311884)
Supplement: S1 File — (ZIP) [file pone.0311884.s001.zip › Supporting information/S1 Fig.pptx]

## Slide 1
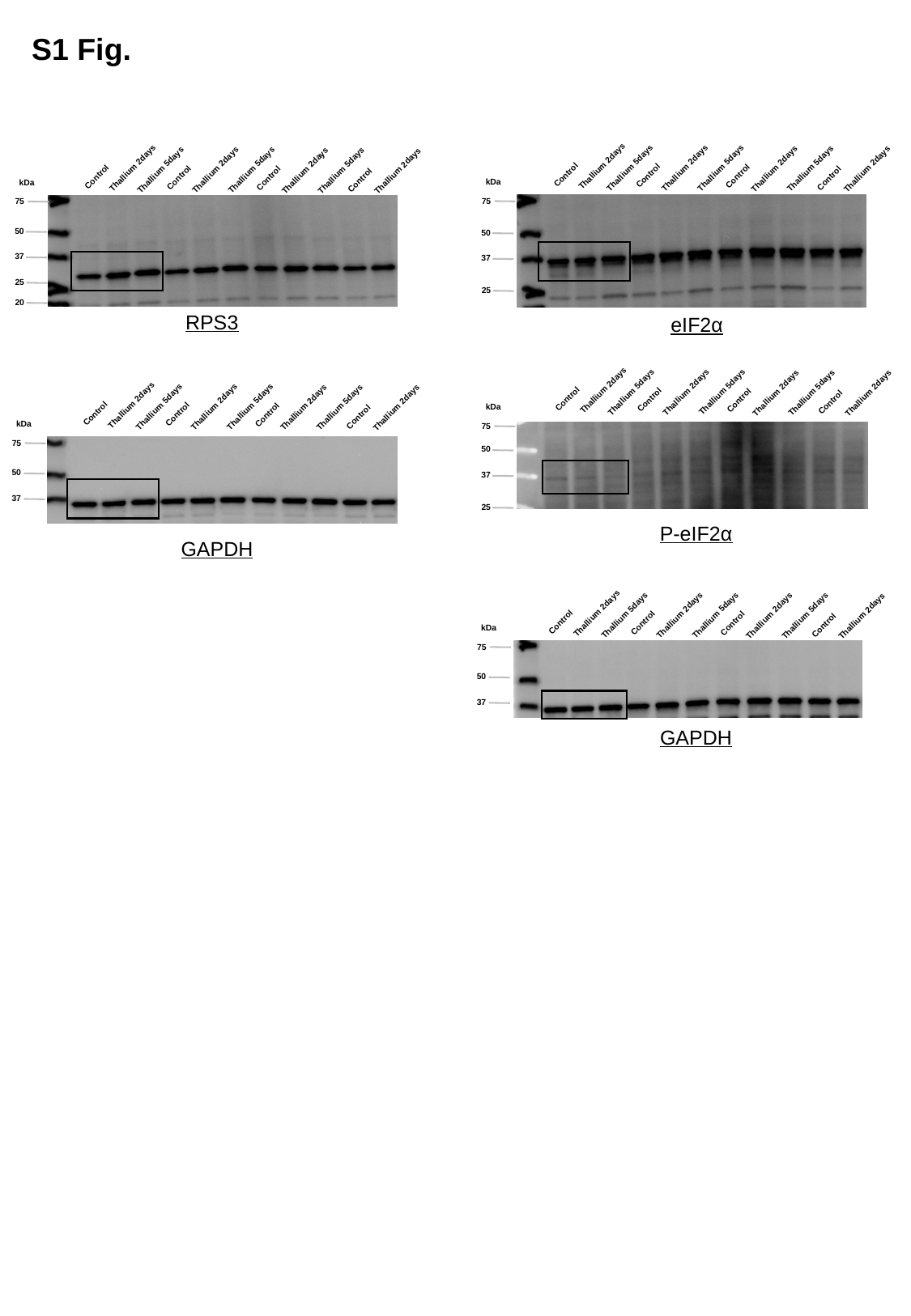

S1 Fig.
Thallium 2days
Thallium 5days
Thallium 2days
Thallium 5days
Thallium 2days
Thallium 5days
Thallium 2days
Thallium 2days
Thallium 5days
Thallium 2days
Thallium 5days
Thallium 5days
Thallium 2days
Thallium 2days
Control
Control
Control
Control
Control
Control
Control
Control
kDa
kDa
75
75
50
50
37
37
25
25
20
RPS3
eIF2α
Thallium 2days
Thallium 5days
Thallium 2days
Thallium 5days
Thallium 5days
Thallium 2days
Thallium 2days
Control
Control
Control
Control
Thallium 2days
Thallium 5days
Thallium 2days
Thallium 5days
Thallium 5days
Thallium 2days
Thallium 2days
kDa
Control
Control
Control
Control
kDa
75
75
50
50
37
37
25
P-eIF2α
GAPDH
Thallium 2days
Thallium 5days
Thallium 2days
Thallium 5days
Thallium 5days
Thallium 2days
Thallium 2days
Control
Control
Control
Control
kDa
75
50
37
GAPDH

## Slide 2
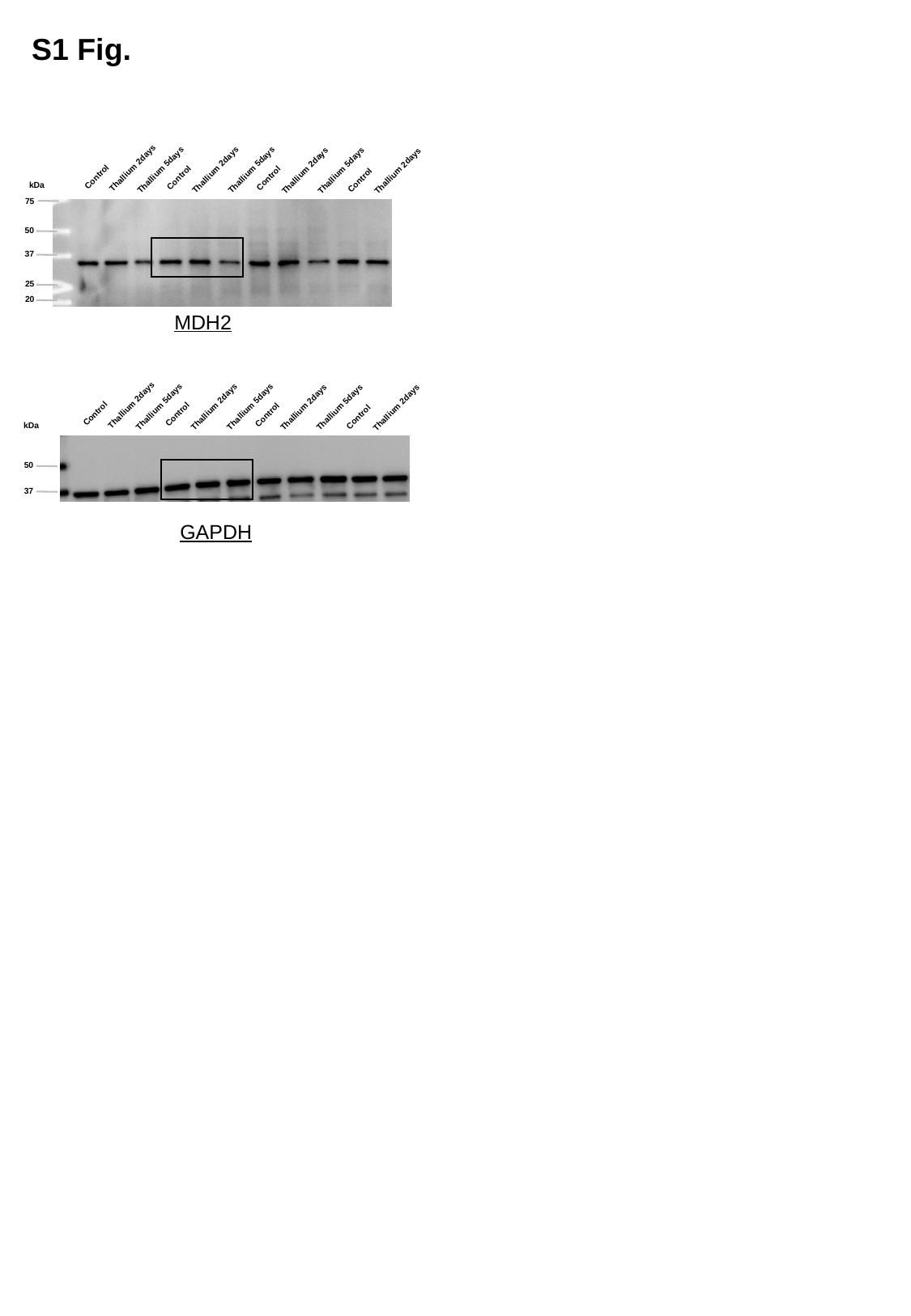

S1 Fig.
Thallium 2days
Thallium 5days
Thallium 2days
Thallium 5days
Thallium 5days
Thallium 2days
Thallium 2days
Control
Control
Control
Control
kDa
75
50
37
25
20
MDH2
Thallium 2days
Thallium 5days
Thallium 2days
Thallium 5days
Thallium 5days
Thallium 2days
Thallium 2days
Control
Control
Control
Control
kDa
50
37
GAPDH

## Slide 3
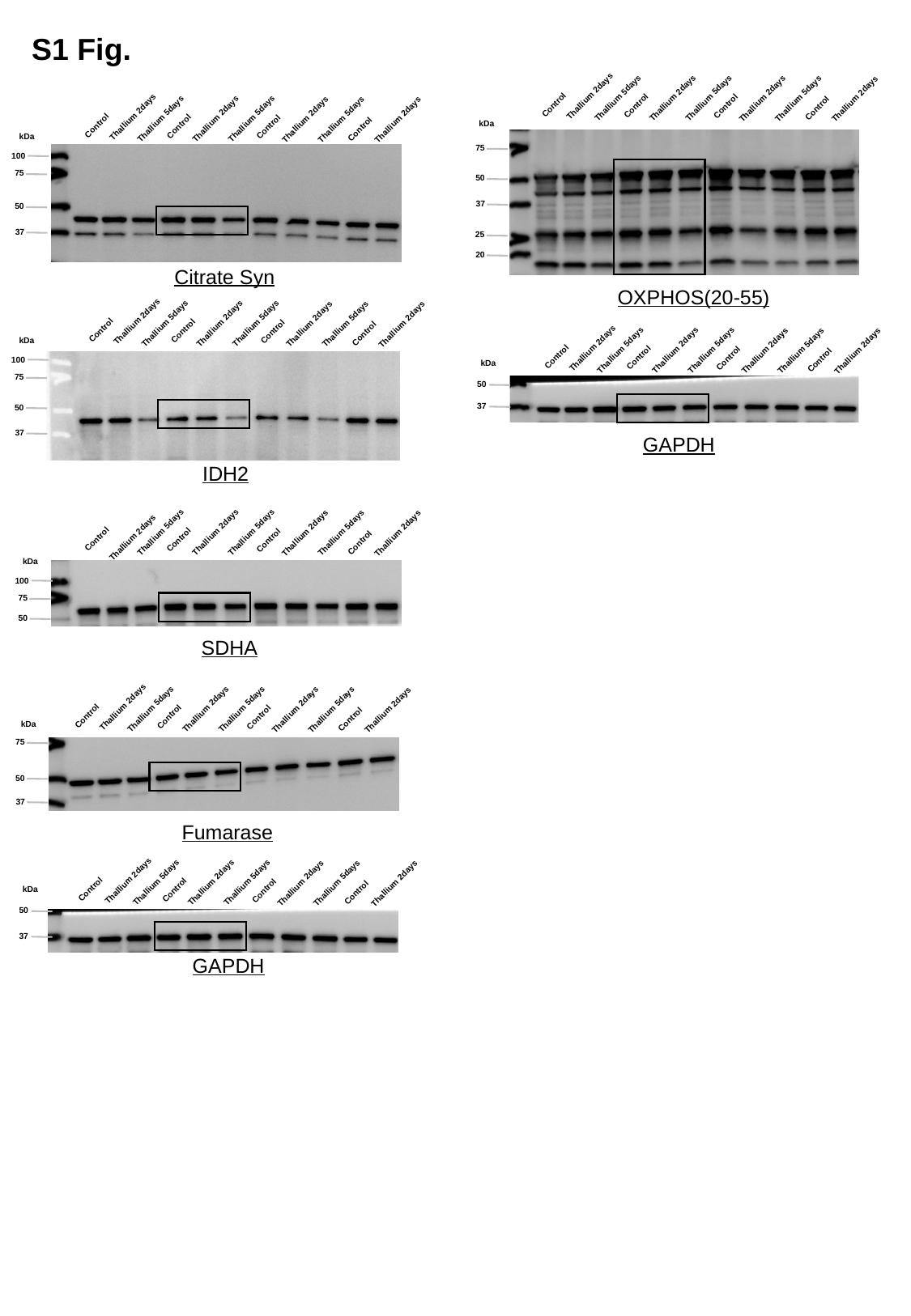

S1 Fig.
Thallium 2days
Thallium 5days
Thallium 2days
Thallium 5days
Thallium 5days
Thallium 2days
Thallium 2days
Control
Control
Control
Control
Thallium 2days
Thallium 5days
Thallium 2days
Thallium 5days
Thallium 5days
Thallium 2days
Thallium 2days
kDa
Control
Control
Control
Control
kDa
75
100
75
50
37
50
37
25
20
Citrate Syn
OXPHOS(20-55)
Thallium 2days
Thallium 5days
Thallium 2days
Thallium 5days
Thallium 5days
Thallium 2days
Thallium 2days
Control
Control
Control
Control
kDa
Thallium 2days
Thallium 5days
Thallium 2days
Thallium 5days
Thallium 5days
Thallium 2days
Thallium 2days
Control
Control
Control
100
Control
kDa
75
50
37
50
37
GAPDH
IDH2
Thallium 5days
Thallium 2days
Thallium 5days
Thallium 5days
Thallium 2days
Thallium 2days
Thallium 2days
Control
Control
Control
Control
kDa
100
75
50
SDHA
Thallium 2days
Thallium 5days
Thallium 2days
Thallium 5days
Thallium 5days
Thallium 2days
Thallium 2days
Control
Control
Control
Control
kDa
75
50
37
Fumarase
Thallium 2days
Thallium 5days
Thallium 2days
Thallium 5days
Thallium 5days
Thallium 2days
Thallium 2days
Control
kDa
Control
Control
Control
50
37
GAPDH

## Slide 4
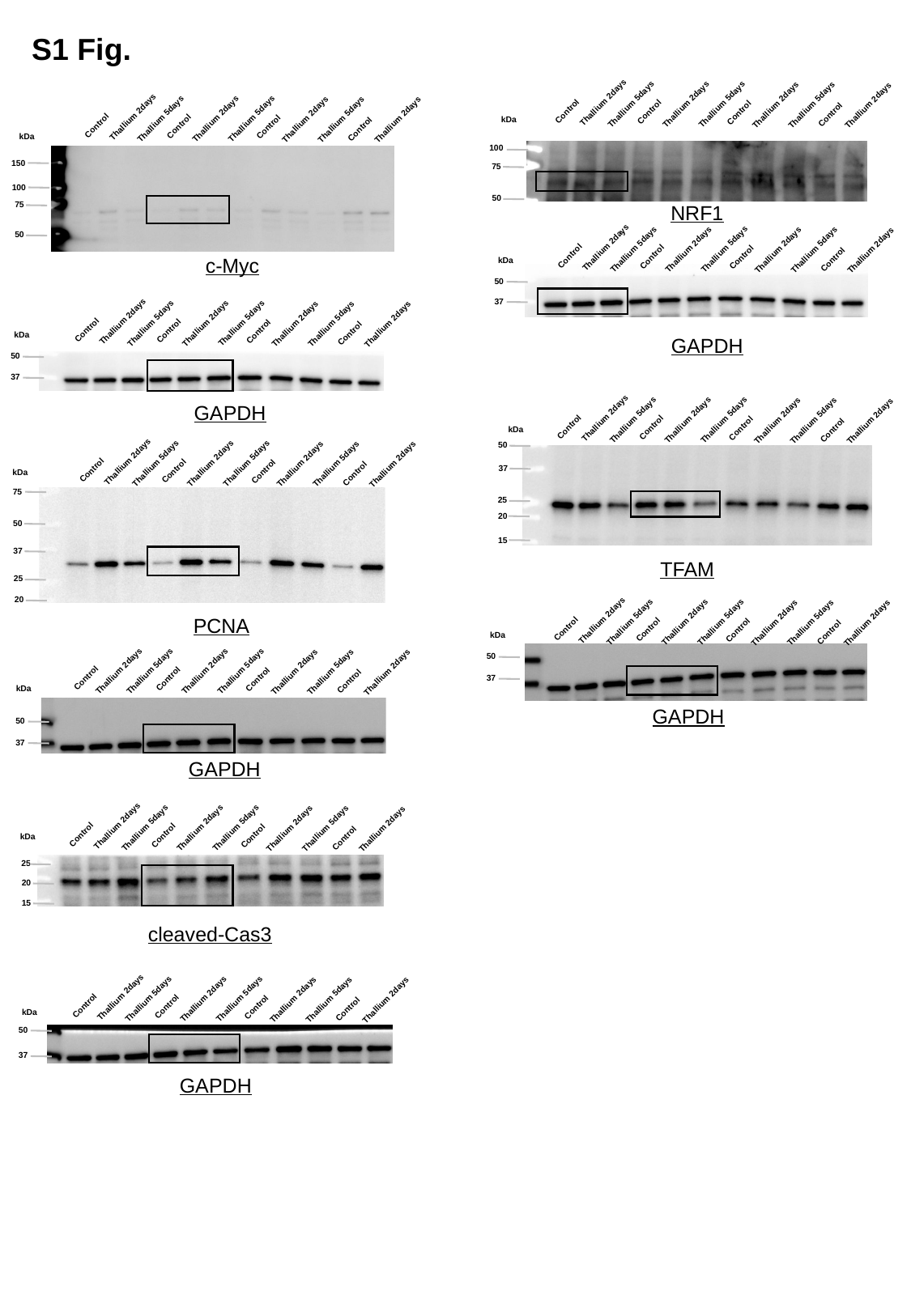

S1 Fig.
Thallium 2days
Thallium 5days
Thallium 2days
Thallium 5days
Thallium 5days
Thallium 2days
Thallium 2days
Control
Control
Control
Control
Thallium 2days
Thallium 5days
Thallium 2days
Thallium 5days
Thallium 5days
Thallium 2days
Thallium 2days
kDa
Control
Control
Control
Control
kDa
100
150
75
100
50
75
NRF1
50
Thallium 2days
Thallium 5days
Thallium 2days
Thallium 5days
Thallium 5days
Thallium 2days
Thallium 2days
Control
Control
Control
c-Myc
Control
kDa
50
37
Thallium 2days
Thallium 5days
Thallium 2days
Thallium 5days
Thallium 5days
Thallium 2days
Thallium 2days
Control
Control
Control
Control
kDa
GAPDH
50
37
GAPDH
Thallium 2days
Thallium 5days
Thallium 2days
Thallium 5days
Thallium 5days
Thallium 2days
Thallium 2days
Control
Control
Control
kDa
Control
50
Thallium 2days
Thallium 5days
Thallium 2days
Thallium 5days
Thallium 5days
Thallium 2days
Thallium 2days
37
Control
Control
Control
kDa
Control
75
25
20
50
15
37
TFAM
25
20
PCNA
Thallium 2days
Thallium 5days
Thallium 2days
Thallium 5days
Thallium 5days
Thallium 2days
Thallium 2days
Control
Control
Control
Control
kDa
50
Thallium 5days
Thallium 2days
Thallium 2days
Thallium 5days
Thallium 5days
Thallium 2days
Thallium 2days
Control
37
Control
Control
Control
kDa
GAPDH
50
37
GAPDH
Thallium 2days
Thallium 5days
Thallium 2days
Thallium 5days
Thallium 5days
Thallium 2days
Thallium 2days
Control
Control
Control
kDa
Control
25
20
15
cleaved-Cas3
Thallium 2days
Thallium 5days
Thallium 2days
Thallium 5days
Thallium 5days
Thallium 2days
Thallium 2days
Control
Control
Control
Control
kDa
50
37
GAPDH
